# Impact of Home Smoking Rules on Smoking Patterns Among Adolescents and Young Adults

**Published:** 2006-03-15

**Authors:** Pamela I Clark, Michael W Schooley, Bennett Pierce, Jane Schulman, Carol L Schmitt, Anne M Hartman

**Affiliations:** Battelle Centers for Public Health Research and Evaluation; Centers for Disease Control and Prevention, Atlanta, Ga; Battelle Memorial Institute, Columbus, Ohio; Battelle Memorial Institute, Crystal City, Va; Battelle Centers for Public Health Research and Evaluation, Baltimore, Md; Risk Factor Monitoring and Methods Branch, Division of Cancer Control and Population Sciences, National Cancer Institute, Bethesda, Md

## Abstract

**Introduction:**

Smoking restrictions in public places have been shown to reduce cigarette consumption and may reduce smoking prevalence. Evidence is emerging that smoke-free policies in nonpublic places may have a similar effect. The purpose of this study was to determine whether an association exists between household smoking rules and smoking patterns among adolescents (aged 15 to 18 years) and young adults (aged 19 to 24 years) living in parental homes (i.e., the homes of their parents, grandparents, or foster parents).

**Methods:**

Cross-sectional data from the 1998–1999 Tobacco Use Supplement to the Current Population Survey were analyzed for the association between household smoking rules and smoking behaviors among adolescents and young adults. We used a probability sample of noninstitutionalized adolescents (aged 15 to 18 years) and young adults (aged 19 to 24 years) living in the United States and assessed smoking status, attempts to quit, and smoking intensity.

**Results:**

After controlling for smoking status of others in the household, the odds of ever having smoked, being a current smoker, and smoking more than five cigarettes per day were significantly smaller in households with strict no-smoking policies than in households where smoking was permitted anywhere. These results were relevant for adolescents and young adults.

**Conclusion:**

Household smoking rules are a type of antitobacco socialization that help deter adolescents from smoking. The influence of household smoking rules seems to extend beyond adolescence into the young adult years among people who continue to live at home with their parents, grandparents, or foster parents.

## Introduction

Smoking bans in public places, whether mandated or voluntary, are effective methods for reducing people's exposure to secondhand smoke (1). In addition to protecting nonsmokers from involuntary exposure to tobacco smoke toxins, such policies reduce cigarette smoking and may increase quitting rates among adult smokers (1-11). For example, workplace smoking bans reduce smoking prevalence by approximately 10% (6) and reduce cigarette smoking by 29% (12). Restrictions on smoking in public places also produce environments in which smoking is marginalized (13).

Smoking bans in nonpublic places, such as homes and cars, have also been associated with reduced smoking among adults (14-16). In addition, nonsmoking environments have been shown to help previous smokers keep from starting to smoke again (15). Home smoking bans are also associated with longer quit attempts among adults (14). A longitudinal study performed in Oregon found that complete household smoking bans doubled the odds of a repeat quit attempt and decreased relapse rates (16). However, studies that conclude that adult smoking patterns are influenced by home smoking bans may be limited if adults who are inclined to quit smoking are also inherently inclined to impose home smoking restrictions. Emerging studies of the effect of home smoking bans on adolescent and young adult smoking may not have the same limitation because adolescents and young adults are less likely to self-impose household smoking bans.

Home smoking bans have been associated with lower smoking prevalence among adolescents. For example, a study based on national surveys of adolescents aged 15 to 17 years conducted between 1992 and 1996 found that adolescents who lived with at least one smoker in households with smoking bans were less likely to be smokers than those living with a smoker in households without smoking bans. Smoking prevalence was lowest among adolescents who lived in households with smoking bans in which no members had ever smoked. In addition, adolescents who had a history of smoking were more likely to have quit if they lived in smoke-free homes (17). In another national study, Wakefield et al (18) found that household smoking bans reduced initiation and transition to regular smoking. In a regional study, Proescholdbell et al (19) found that middle school and high school students with restrictive home smoking policies were less likely to begin smoking. However, Biener et al (20) found no association between household smoking policies and regular smoking among middle school students.

Although most people who smoke begin doing so before age 18 (21), young adults (aged 18–25 years) are still more likely than older adults (aged >25 years) to begin smoking, transition to regular smoking, and be targeted by tobacco industry marketing (22). In fact, the regular smoking rates of young adults are equal to the rates of adults aged 25 to 44 years, which are typically the highest rates overall (22,23). One previous investigation into the effects of residential smoking policies on young adult smoking found lower smoking rates among college students living in smoke-free housing (24).

The purpose of this study was to determine whether an association exists between household smoking rules and smoking patterns among adolescents and young adults living in *parental homes* — the homes of their parents, grandparents, or foster parents. The analysis was restricted to youths who live in parental homes because they are primarily influenced by rules that have been established by other people. The study focused on adolescents and young adults aged 15 to 24 to expand the previously reported age range results. We used data from the 1998–1999 Tobacco Use Supplement to the Current Population Survey (TUS–CPS) because it included adolescent and young adult respondents and allowed us to assess the impact of home smoking rules on various tobacco use measures.

## Methods

The CPS is a national probability-based survey that has been conducted monthly for the past 50 years by the U.S. Census Bureau and the U.S. Bureau of Labor Statistics (25). The CPS uses a household-based sampling frame in which households are selected to participate based on their geographic location to ensure samples are representative of the entire United States, individual states, and other geographic entities such as the District of Columbia. Within each sampled household, noninstitutionalized individuals aged 15 years and older are eligible to participate. Core CPS questions are occasionally supplemented with questions on specific subjects, such as those in the TUS. Our results are based on responses to the September 1998, January 1999, and May 1999 TUS–CPS (25).

Approximately 48,000 households with about 95,000 potential respondents were included in the sample for each of these monthly surveys. The core CPS household response rate was about 93%. Response rates for the TUS among households already responding to the core CPS were 81.8% for September 1998, 84.4% for January 1999, and 81.8% for May 1999, yielding approximately 80,000 completed interviews in each survey.

We divided the TUS-CPS data into a subset that included respondents aged 15 to 24 years who lived in parental homes (i.e., with a parent, grandparent, or foster parent). Although proxy responses were permitted for selected questions on the TUS, including smoking status and the use of other tobacco products, we restricted this analysis to self-reporting respondents who answered the questions, "Have you smoked at least 100 cigarettes in your entire life?" and "Which statement best describes the rules about smoking in your home?" About 80% of the TUS responses were self-responses. Of the 25,208 individuals aged 15 to 24 residing in a parental home with an adult head of household who were interviewed for the TUS-CPS, 49.2% (12,408) self-responded to the supplement. Of the supplement self-respondents for whom an interview was conducted, 99.1% (12,299) of the respondents also provided information on home smoking bans and other relevant variables for the analysis.

### Measures

Smoking outcomes were developed for the following: age of smoking initiation, smoking status, smoking intensity, quit attempts, and cessation (Table 1). The primary predictor variable — home smoking rules — was based on responses to the question, "What statement best describes the rules about smoking in your home?" Three response categories were used: 1) "no one is allowed to smoke anywhere," 2) "smoking is allowed in some places or at some times," and 3) "smoking is permitted anywhere." The smoking status of other household members in addition to several demographic variables, including the respondent's age, sex, race or ethnicity, and household income, were considered additional covariates because of their association with the smoking outcome variables.

### Statistical analyses

Frequency distributions for the variables were examined, and the relationships between home smoking rules and the various smoking measures and covariates were explored. A series of statistical models was developed to examine the associations between home smoking rules and the outcomes of interest. Separate main effects models that included home smoking rules, age, sex, race or ethnicity, household income, and the smoking status of other household members as categorical variables were created for each smoking outcome variable. All covariates were retained in each model for consistency and to control for residual confounding. Interaction terms between home smoking rules and race or ethnicity, age, and smoking status of other household members were investigated individually, but the results were not included because they were not statistically significant and did not meaningfully change the modeling results.

A linear regression model was used to assess the relationship between home smoking rules and age of initiation. Logistic regression models with a logit link were used for dichotomous outcomes, such as whether respondents had ever smoked 100 or more cigarettes in their lifetime (yes or no) or had ever attempted to quit smoking (yes or no). Polytomous logistic regression models with generalized logits were used for categorical outcomes that had more than two response levels, such as the number of cigarettes smoked per day. SUDAAN (Research Triangle Institute, Research Triangle Park, NC) was used to account for the complex sample design of the CPS. Balance repeated replication methods with replicate weights and Fay's perturbation factor (26) of 0.5 were used for variance estimation. Weights and replicate weights associated with self-reported responses were also used.

## Results

The response distribution is shown in Table 2. In response to the question about home smoking rules, 62.0% answered that "no one is allowed to smoke anywhere in the household," and the remaining respondents were approximately evenly divided between "smoking is allowed in some places or at some times" (20.0%) and "smoking is permitted anywhere" (18.0%).

Preliminary examination of the covariates (excluding proxy responses) indicated that the smoking status of other household members was unknown for 39.7% of the respondents; the remaining 60.3% of the responses were fairly equally divided between "no one else in the household smokes" (28.9%) and "at least one other person in the household smokes" (31.4%).

In response to the question about whether they had ever smoked, 17.7% of the respondents reported that they had smoked 100 or more cigarettes in their lifetime, and 13.9% reported that they were current smokers (i.e., smoked some days or every day). The average age of smoking initiation among the sampled respondents was 15 years. The number of cigarettes smoked per day was usually reported using the break points of one-fourth, one-half, and one pack (20 cigarettes). Of the current smokers, 31.1% smoked 5 or fewer cigarettes per day, 28.9% smoked 6 to 10 cigarettes per day, 34.6% smoked 11 to 20 cigarettes per day, and 5.4% smoked more than a pack per day. Of the current smokers, 46.1% had tried to quit for 1 day or longer, and 59.7% of former smokers had quit for more than 180 days.

The exploratory analysis indicated that home smoking rules (either not being allowed to smoke anywhere or being allowed to smoke in some places or at some times) were associated with ever having smoked, current smoking, smoking intensity, and quit ratio (the proportion of people who had ever smoked who reported that they were former smokers at the time of the interview) (Table 3). Furthermore, the effects were greater in households with strict smoking rules (no one allowed to smoke anywhere) than in households with some smoking rules (smoking allowed in some places or at some times). The prevalence of people who had ever smoked was 2.5 times higher in households without smoking rules (30.3%) and 2 times higher in households with some smoking rules (24.0%) than in households where no smoking was allowed at all (12.0%). The prevalence of current smokers was approximately 3 times higher in households without smoking rules (26.5%) and approximately 2 times higher in households with some smoking rules (19.2%) than in households where no smoking was allowed at all (8.5%).

A higher smoking intensity was found in households without home smoking rules than in households with home smoking rules. In households without home smoking rules, 49.7% of the respondents reported smoking more than 10 cigarettes per day, compared with 38.7% of respondents in households with some smoking rules and 32.2% of respondents in households with strict smoking rules.

More respondents in households with home smoking rules reported having quit smoking than in households without home smoking rules. The quit ratio was more than twice as large in households where no one was allowed to smoke (28.9%) than in households without smoking rules (12.5%). No statistically significant association was found between home smoking rules and age of initiation, ever trying to quit, the number of quit attempts in the previous year, and whether fewer or more than 180 days had elapsed since quitting.

The results from the multivariate analysis are shown in Table 4. Home smoking rules were significantly associated with ever having smoked 100 cigarettes or more, being a current smoker, smoking intensity, and having quit smoking. For example, after controlling for sex, age, household income, race or ethnicity, and smoking status of other household members, the adjusted odds of ever having smoked 100 or more cigarettes were significantly lower in households with strict smoking rules (i.e., where no one can smoke anywhere) than in households without any smoking rules (i.e., where anyone can smoke anywhere) (odds ratio [OR] = 0.56; 95% confidence interval [CI],  0.47–0.66). The adjusted odds of being a current smoker compared with never having smoked were approximately 50% lower in households with strict smoking rules than in households without any smoking restrictions (OR = 0.48; 95% CI, 0.39–0.57). The adjusted odds of smoking six or more cigarettes per day compared with five or fewer were significantly lower in households with strict smoking rules than in households without smoking rules (OR = 0.40; 95% CI, 0.28–0.59). The adjusted odds of having quit were more than 2 times higher in households with strict smoking rules than in households without smoking rules. Similarly, the adjusted odds of having quit smoking were about 60% higher in households with some smoking rules than in households without smoking rules. No other significant results were found in households with some smoking rules compared with households without smoking rules.

Table 4 shows the similarity between adolescents and young adults. In both groups, strict smoking rules were found to be associated with never having smoked 100 cigarettes or more, not being a current smoker, and having quit smoking, although the odds of having quit smoking were of borderline significance for adolescents. The adjusted odds of smoking more than six cigarettes per day (compared with five or fewer) were significantly lower for both groups, although the adjusted odds of smoking more than 10 cigarettes per day were significantly lower only for the young adults.

## Discussion

Smoking restrictions at work, in schools, and in other public places have been associated with reduced smoking prevalence among adults, and reports of their influence on adolescents are emerging (17-19). Our study is the first to report an association between home smoking rules and reduced smoking among adolescents and young adults.

This study provides valuable insight into parental influences on the smoking behaviors of young adults and adolescents. Based on a national probability sample of households, we found that among adolescents and young adults aged 15 to 24 years and living at home, the odds of ever having smoked 100 cigarettes or more, being a current smoker, and smoking more than five cigarettes per day were significantly lower in households with strict smoking rules than in households without smoking rules. The odds of having quit smoking were also significantly higher in households with strict or some smoking rules than in households without any smoking rules.

Our findings reinforce the theory that household smoking rules can be among many parental antismoking measures that collectively result in antitobacco socialization of adolescents and young adults. Even in homes in which parents smoke, prohibiting youths from smoking and clearly communicating smoking rules have been shown to significantly reduce smoking initiation, stage of uptake (i.e., progression from experimentation to regular use), and current smoking and increase smoking cessation rates (27-33). Household smoking bans, such as the strict smoking rules considered in our study, that apply to all people (including adults) living in and visiting a home could be a powerful form of antitobacco socialization. When youths see that adults must leave the house to smoke, it sends a clear message that smoking is not condoned; allowing adults to smoke in the home sends the opposite message (17).

Establishing and clearly communicating the terms of a household smoking ban may be a parental behavior that is amenable to a public health intervention, such as a communication campaign. Any such intervention would build on a movement that already has momentum — reducing indoor smoking to protect children from environmental tobacco smoke. The prevalence of environmental tobacco smoke exposure in the homes of children aged younger than 18 years decreased from 35.6% to 25.1% (*P* < .001) between 1992 and 2000, a decrease that is higher than would be predicted by the decrease in smoking prevalence (26.5% in 1992 to 23.3% in 2000) (34). In addition, public acceptance of smoke-free environments is growing (13). It seems logical that promoting another reason for establishing household smoking rules (i.e., other than to decrease exposure to environmental tobacco smoke) — which is to decrease smoking among adolescents and young adults — would contribute to the smoking decrease in households with children.

Ours is the first report to suggest that the effects of home smoking rules on adolescents may persist in young adulthood among those living in parental homes. Our finding that restrictive home smoking rules have similar effects on adolescents and young adults is important given current smoking rates among young adults. The U.S. prevalence of current smoking in 2002 was found to be highest among people aged 18 to 24 years (28.5%; 95% CI, 26.5%–30.5%), which is statistically equal to the 25.7% (95% CI, 24.7%–26.7%) among adults aged 25 to 44 years — the age group with the highest smoking rates (23,35). If a cohort effect contributed to the increase in smoking among young adults, the results may not be generalizable to other cohorts. Between 1991 and 1997, an increase in smoking was found among adolescents, so the aging of the group into the population of young adults may have contributed to the accompanying increase in smoking among young adults. However, this cohort effect is unlikely to explain the overall high smoking rate among young adults because the increase in smoking among 18- to 24-year-olds began just before the increase in smoking among high school seniors (36). If people were becoming established smokers at older ages, this, too, would result in higher smoking rates among young adults. For example, data from the National Health Interview Survey show that people are becoming regular smokers when they are ages 19 to 21 years, rather than before age 18 as they were in previous cohorts (36). Only 13.5% of the 1974 birth cohort reported becoming regular smokers after age 18, compared with 17.8% of the 1975 birth cohort and 21.7% of the 1977 cohort (36). Also contributing to the higher smoking rate among young adults may be concerted marketing by tobacco companies that target this age group (37,38).

Regardless, we know little about the causes of the upward trend in smoking in the young adult group and even less about ways to prevent the initiation of and increase in smoking among young adults. Previous researchers of tobacco use among young adults have only reported the effects of residential smoking rules on smoking among college students. Wechsler et al (24) found that the current smoking prevalence was significantly lower among residents of smoke-free housing (21.0%) than among residents of housing without smoking restrictions (30.6%; *P* < .001), although the effect was only found among students who had not been regular smokers before age 19 years (10% compared with 16.9%; *P* < .001) and was not found among students who become regular smokers during adolescence.

Interpretation of our study's findings is complicated. To ensure that the sampled respondents were primarily being influenced by rules set by others, the analyses were restricted to people who lived in homes with at least one adult head of household. Young adults living in parental  homes may be influenced by peers and environmental factors differently than young adults living outside of parental homes. Because the TUS-CPS is a cross-sectional survey, we do not know whether establishing strict home smoking rules helps prevent youths from smoking or whether youths who are already less likely to smoke are also more likely to live in a home with established smoking rules. In addition, self-reports are inherently limited but would not produce significant bias unless one group experienced differential underreporting compared with another. We do not know whether youths living in households with strict smoking restrictions are less likely to be honest about their smoking status than youths living in households with less restrictive rules. In addition, a classification error may be associated with home smoking rules. Another study using the TUS-CPS data, which reported home smoking rules by multiple household members, reported that approximately 12% of the responses were discrepant (35). This finding may reflect the informal nature of some household rules and the failure of family members to talk explicitly about the rules. For instance, in a study of setting rules about adolescent smoking, approximately 50% of parents who reported having such rules also reported not having informed their adolescent children of the rules (27). It may be expected that households in which no adults smoked would be less likely to clarify the rules against smoking. However, it was found that discrepancies in reporting household smoking policies occurred more often in households with children and in households with adult smokers (35).

It may seem that our finding of only 12.9% current smoking prevalence among adolescents and young adults is unusually low. However, the TUS-CPS limited us to the adult definition of current smoking. In other words, we could not determine who had smoked in the past month, and the question "Have you ever smoked at least 100 cigarettes in your entire life" was used to categorize people as being "ever smokers" or "never smokers." In addition, young adults living in parental homes may be different from young adults who are living outside the home or in college housing. However, the smoking prevalence rate for the entire sample of adolescents (9.9%) and young adults (21.9%) in the TUS-CPS sample was not substantially different from the rate for those living in parental homes.

Community norms that marginalize smoking and results of other smoking-control interventions could influence the prevalence of household smoking restrictions. Future studies leading to an understanding of the influences of community norms and other possible determinants of household restrictions would be helpful in planning public health interventions.

Overall, we found that strict household smoking bans, compared with partial bans, reduced the odds of ever having smoked, being a current smoker, and smoking more than five cigarettes per day among adolescents and young adults living in parental homes. Therefore, homes with strict no-smoking rules not only protect youths from exposure to environmental tobacco smoke but also affect their smoking behaviors. Public health practice should include interventions that encourage the establishment of smoke-free homes.

## Figures and Tables

**Table 1:** Smoking Patterns Among Adolescents and Young Adults: Outcome Variables, Indicator Variables, and Covariates^a^

**Variable**	**Item**	**Category**

**Outcome variable**		

Age of initiation	How old were you when you first started smoking cigarettes fairly regularly?	Numeric: 7-24 years of age, inclusive
Smoking status	Have you smoked at least 100 cigarettes in your entire life?	Yes = ever smokerNo = never smoker
	Do you now smoke cigarettes every day, some days, or not at all?	Among ever smokers: Every day or some days = current smoker Not at all = former smoker
Smoking intensity	On average, how many cigarettes do you now smoke a day?	Among respondents who smoke every day, average number of cigarettes smoked per day:≤5 6-10 11-15 16-20≥21
	On how many of the past 30 days did you smoke? How many cigarettes do you smoke a day?	Among smokers who smoke on some days but not every day, average number of cigarettes smoked per day:≤5 6-10 11-15 16-20≥21
Quit attempts	Have you ever stopped smoking for 1 day or longer because you are trying to quit smoking?	Among current smokers: Yes = ever attempted >No = never attempted
	How many times during the last 12 months have you stopped smoking for 1 day or longer because you were trying to quit smoking?	Among current smokers, number of quit attempts in last 12 months:None 1 2 3≥4
Having quit	About how long has it been since you completely stopped smoking cigarettes?	Among former smokers:<180 or ≥180 days

**Indicator variable**		

Home smoking rules	What statement best describes the rules about smoking in your home?	No one is allowed to smoke anywhere. Smoking is allowed in some places or at some times. Smoking is permitted anywhere.

**Covariate**		

Age, y	Respondent's age at of end of survey week	15-1617-1819-24
Sex	Respondent's sex as recorded during interview	MaleFemale
Race or ethnicity	What is your race? What is your origin or descent?	Non-Hispanic whiteNon-Hispanic blackHispanicOther
Annual household income	Which category represents the total combined income?	<$20,000 $20,000-$39,999 $40,000-$59,999 ≥$60,000
Smoking status of other household members	Based on calculated smoking status defined in previous section for all members in the household; response classified as *unknown* if other interviewed household members were nonsmokers and there were still uninterviewed household members for which smoking status could not be ascertained.	At least one other person in household smokes. No other person smokes. Unknown whether anyone else in household smokes.

a Based on data from the 1998–1999 Tobacco Use Supplement to the Current Population Survey. Includes self-reporting respondents aged 15–24 years.

**Table 2:** Smoking Patterns Among Adolescents and Young Adults: Response Distribution of Outcome Variables, Indicator Variable, and Covariates^a^

**Variable**	**% (95% CIb^b^)**

**Outcome variable**	

**Ever smoked 100 or more cigarettes**	
Yes	17.7 (16.9-18.5)
No	82.3 (81.5-83.1)
**Age of initiation, y**	
≤13	18.5 (16.4-20.5)
14	13.6 (11.7-15.5)
15	16.7 (14.8-18.6)
16	19.7 (17.6-21.8)
17	14.4 (12.8-16.1)
≥18	17.1 (15.3-18.9)
**Smoking status**	
Current smoker	13.9 (13.2-14.6)
Former smoker	3.8 (3.3-4.3)
Never smoker	82.3 (81.5-83.1)
**Number of cigarettes smoked per day**	
≤5	31.1 (28.7-33.5)
6-10	28.9 (26.7-31.1)
11-15	11.7 (10.0-13.5)
16-20	22.9 (20.7-25.0)
≥21	5.4 (4.4-6.4)
**Ever tried to quit for 1 day or longer**	
Yes	46.1 (43.3-48.9)
No	53.9 (51.1-56.7)
**Number of quit attempts in previous 12 months**	
0	53.6 (50.3-56.9)
1	14.2 (11.6-16.9)
2	13.2 (11.2-15.2)
3	8.2 (6.6-9.8)
≥4	10.8 (8.6-13.0)
**Time since quit**	
Quit for ≥180 days	59.7 (55.3-64.1)
Quit for <180 days	40.3 (35.9-44.7)

**Indicator variable**	

**Home smoking rules**	
No one is allowed to smoke anywhere	62.0 (61.0-63.1)
Smoking is allowed in some places or at some times	20.0 (19.2-20.7)
Smoking is permitted anywhere	18.0 (17.3-18.7)

**Covariate**	

**Age, y **	
15-16	35.4 (34.8-35.9)
17-18	31.4 (30.6-32.2)
19-24	33.2 (32.4-34.0)
**Sex**	
Male	52.8 (52.1-53.5)
Female	47.2 (46.5-47.9)
**Race or ethnicity **	
Non-Hispanic white	69.0 (68.3-69.7)
Non-Hispanic black	13.4 (12.8-14.0)
Hispanic	13.6 (13.1-14.2)
Other	4.0 (3.6-4.3)
**Household income **	
<$19,999	23.1 (22.0-24.1)
$20,000-$39,999	23.0 (22.2-23.9)
$40,000-$59,999	20.3 (19.3-21.2)
≥$60,000	33.7 (32.7-34.7)
**Smoking status of other household members**	
At least one other person in the household smokes	31.4 (30.5-32.3)
No one else in the household smokes	28.9 (27.8-29.9)
Unknown whether anyone else in the household smokes	39.7 (38.6-40.9)

a Based on data from the 1998–1999 Tobacco Use Supplement to the Current Population Survey. Includes self-reporting respondents aged 15–24 years

b CI indicates confidence interval.

**Table 3:** Smoking Patterns Among Adolescents and Young Adults: Smoking History Categorized by Home Smoking Rules^a^

**Smoking History**	**Home Smoking Rules**		

	**No One Is Allowed to Smoke Anywhere,** **% (95% CI^b^)**	**Smoking Is Allowed in Some Places or at Some Times,** **% (95% CI^b^)**	**Smoking is Permitted Anywhere** **%, (95% CI^b^)**
**Ever smoked 100 or more cigarettes (yes)**	12.0 (11.2-12.9)	24.0 (22.0-26.0)	30.3 (28.1-32.4)
**Smoking status**			
Current	8.5 (7.8-9.3)	19.2 (17.2-21.2)	26.5 (24.5-28.5)
Former	3.5 (2.9-4.0)	4.8 (3.8-5.7)	3.8 (2.9-4.7)
Never	88.0 (87.1-88.8)	76.0 (74.0-78.0)	69.7 (67.6-71.9)
**Smoking intensity (no. cigarettes/day)**			
≤5	40.1 (35.7-44.4)	31.9 (27.0-36.9)	20.6 (16.3-24.8)
6-10	27.8 (23.9-31.7)	29.4 (24.6-34.1)	29.7 (25.2-34.2)
>10	32.2 (27.6-36.7)	38.7 (33.5-43.9)	49.7 (45.0-54.5)
**Quit ratio^c^**	28.9 (25.2-32.6)	19.9 (16.1-23.8)	12.5 (9.9-15.2)

a Based on data from the 1998–1999 Tobacco Use Supplement to the Current Population Survey. Includes self-reporting respondents aged 15–24 years

b CI indicates confidence interval.

c The proportion of ever smokers who reported that they were former smokers at the time of the interview.

**Table 4:** Comparison of Smoking Patterns Among Adolescents and Young Adults According to Home Smoking Rules^a^

**Outcome Measures**	**Home Smoking Rules**	

	**No One Is Allowed to Smoke^b^**	**Smoking Is Allowed in Some Places or Some Times^b^**

	**Adjusted Odds Ratio(95% CI^c^)**	**Adjusted Odds Ratio(95% CI^c^)**

**Ever smokers^d^(compared with never smokers) **		

Ages 15-24 y	0.56 (0.47-0.66)	0.99 (0.84-1.17)
Ages 15-18 y	0.56 (0.44-0.71)	0.99 (0.78-1.28)
Ages 19-24 y	0.56 (0.45-0.70)	0.99 (0.78-1.26)

**Current smokers(compared with never smokers)**		

Ages 15-24 y	0.48 (0.39-0.57)	0.92 ( 0.78-1.10)
Ages 15-18 y	0.51 (0.40-0.67)	0.98 (0.76-1.27)
Ages 19-24 y	0.45 (0.36-0.58)	0.88 (0.68-1.14)

**Current smokers among ever smokers^d^(compared with former smokers among ever smokers)**		

Ages 15-24 y	0.44 (0.32-0.60)	0.62 (0.45-0.85)
Ages 15-18 y	0.64 (0.41-1.00)	0.88 (0.55-1.43)
Ages 19-24 y	0.33 (0.21-0.53)	0.46 (0.29-0.73)

**No. cigarettes smoked per day(compared with smoking 5 or fewer cigarettes per day)**		

**Ages 15-24 y**		
6-10	0.40 (0.28-0.59)	0.66 (0.44-0.99)
>10	0.51 (0.34-0.77)	0.71 (0.45-1.10)
**Ages 15-18 y**		
6-10	0.43 (0.24-0.77)	0.64 (0.36-1.13)
>10	0.67 (0.38-1.16)	1.39 (0.68-2.83)
**Ages 19-24 y**		
6-10	0.40 (0.24-0.67)	0.73 (0.43-1.26)
>10	0.42 (0.24-0.71)	0.87 (0.51-1.48)

a Based on data from the 1998–1999 Tobacco Use Supplement to the Current Population Survey. Includes self-reporting respondents aged 15–24 years.

b Compared with homes in which smoking is permitted anywhere.

c CI indicates confidence interval. Odds ratio adjusted for sex, age, household income, race or ethnicity, and smoking status of other household members.

d Ever smokers were smokers who had smoked 100 or more cigarettes in their lifetime; never smokers were smokers who had smoked fewer than 100 cigarettes in their lifetime.

## References

[1] Task Force on Community Preventive Services (2001). Tobacco use prevention and control. Am J Prev Med.

[2] Biener L, Nyman AL (1999). Effect of workplace smoking policies on smoking cessation: results of a longitudinal study. J Occup Environ Med.

[3] Borland R, Owen N, Tooley G, Treijs I, Roberts L, Hill D (1999). Promoting reduced smoking rates in the context of workplace smoking bans. Am J Health Promot.

[4] Brigham J, Gross J, Stitzer ML, Felch LJ (1994). Effects of a restricted work-site smoking policy on employees who smoke. Am J Public Health.

[5] Chapman S, Borland R, Scollo M, Brownson RC, Dominello A, Woodward S (1999). The impact of smoke-free workplaces on declining cigarette consumption in Australia and the United States. Am J Public Health.

[6] Farrelly MC, Evans WN, Sfekas AE (1999). The impact of workplace smoking bans: results from a national survey. Tob Control.

[7] Glasgow RE, Cummings KM, Hyland A (1997). Relationship of worksite smoking policy to changes in employee tobacco use: findings from COMMIT. Community Intervention Trial for Smoking Cessation. Tob Control.

[8] Gottlieb NH, Eriksen MP, Lovato CY, Weinstein RP, Green LW (1990). Impact of a restrictive work site smoking policy on smoking behavior, attitudes, and norms. J Occup Med.

[9] Kinne S, Kristal AR, White E, Hunt J (1993). Work-site smoking policies: their population impact in Washington State. Am J Public Health.

[10] Stave GM, Jackson GW (1991). Effect of a total work-site smoking ban on employee smoking and attitudes. J Occup Med.

[11] Woodruff TJ, Rosbrook B, Pierce J, Glantz SA (1993). Lower levels of cigarette consumption found in smoke-free workplaces in California. Arch Intern Med.

[12] Fichtenberg CM, Glantz SA (2002). Effect of smoke-free workplaces on smoking behaviour: systematic review. BMJ.

[13] Gilpin EA, Lee L, Pierce JP (2004). Changes in population attitudes about where smoking should not be allowed: California versus the rest of the USA. Tob Control.

[14] Gilpin EA, White MM, Farkas AJ, Pierce JP (1999). Home smoking restrictions: which smokers have them and how they are associated with smoking behavior. Nicotine Tob Res.

[15] Horwitz MB, Hindi-Alexander M, Wagner TJ (1985). Psychosocial mediators of abstinence, relapse, and continued smoking: a one-year follow-up of a minimal intervention. Addict Behav.

[16] Pizacani BA, Martin DP, Stark MJ, Koepsell TD, Thompson B, Diehr P (2004). A prospective study of household smoking bans and subsequent cessation related behaviour: the role of stage of change. Tob Control.

[17] Farkas AJ, Gilpin EA, White MM, Pierce JP (2000). Association between household and workplace smoking restrictions and adolescent smoking. JAMA.

[18] Wakefield M, Chaloupka F, Kaufman N, Orleans C, Barker D, Ruel E (2002). Do restrictions on smoking at home, at school and in public places influence teenage smoking?. ImpacTeen Paper Series.

[19] Proescholdbell RJ, Chassin L, MacKinnon DP (2000). Home smoking restrictions and adolescent smoking. Nicotine Tob Res.

[20] Biener L, Cullen D, Di ZX, Hammond SK (1997). Household smoking restrictions and adolescents' exposure to environmental tobacco smoke. Prev Med.

[21] (1994). U.S. Department of Health and Human Services. Preventing tobacco use among young people: a report of the Surgeon General.

[22] Biener L, Albers AB (2004). Young adults: vulnerable new targets of tobacco marketing. Am J Public Health.

[23] Centers for Disease Control and Prevention (2004). Cigarette smoking among adults — United States, 2002. MMWR Morb Mortal Wkly Rep.

[24] Wechsler H, Lee JE, Rigotti NA (2001). Cigarette use by college students in smoke-free housing: results of a national study. Am J Prev Med.

[25] (1999). National Cancer Institute. Tobacco Use Supplement to the Current Population Survey, 1998–1999 (Internet).

[26] Judkins D (1990). Fay's method for variance estimation. Journal of Official Statistics.

[27] Clark PI, Scarisbrick-Hauser A, Gautam SP, Wirk SJ (1999). Anti-tobacco socialization in homes of African-American and white parents, and smoking and nonsmoking parents. J Adolesc Health.

[28] Farkas AJ, Distefan JM, Choi WS, Gilpin EA, Pierce JP (1999). Does parental smoking cessation discourage adolescent smoking?. Prev Med.

[29] Henriksen L, Jackson C (1998). Anti-smoking socialization: relationship to parent and child smoking status. Health Communication.

[30] Jackson C, Henriksen L (1997). Do as I say: parent smoking, antismoking socialization, and smoking onset among children. Addict Behav.

[31] Jackson C, Dickinson D (2003). Can parents who smoke socialise their children against smoking? Results from the Smoke-free Kids intervention trial. Tob Control.

[32] Sargent JD, Dalton M (2001). Does parental disapproval of smoking prevent adolescents from becoming established smokers?. Pediatrics.

[33] Andersen MR, Leroux BG, Bricker JB, Rajan KB, Peterson AV (2004). Antismoking parenting practices are associated with reduced rates of adolescent smoking. Arch Pediatr Adolesc Med.

[34] Soliman S, Pollack HA, Warner KE (2004). Decrease in the prevalence of environmental tobacco smoke exposure in the home during the 1990s in families with children. Am J Public Health.

[35] Mumford EA, Levy DT, Romano EO (2004). Home smoking restrictions. Problems in classification. Am J Prev Med.

[36] Lantz PM (2003). Smoking on the rise among young adults: implications for research and policy. Tob Control.

[37] Ling PM, Glantz SA (2002). Why and how the tobacco industry sells cigarettes to young adults: evidence from industry documents. Am J Public Health.

[38] Sepe E, Ling PM, Glantz SA (2002). Smooth moves: bar and nightclub tobacco promotions that target young adults. Am J Public Health.

